# Editorial: Educational neuroscience: key processes and approaches to measurement

**DOI:** 10.3389/fpsyg.2023.1342147

**Published:** 2024-01-08

**Authors:** Rebecca Gordon, Roberto A. Ferreira, Cristina Rodriguez, Andrew Tolmie

**Affiliations:** ^1^UCL Institute of Education, University College London, London, United Kingdom; ^2^Facultad de Ciencias de la Educación, Universidad de Talca, Talca, Chile; ^3^Millennium Nucleus for the Science of Learning (MiNSoL), Talca, Chile; ^4^Facultad de Ciencias de la Educatión, Universidad Católica del Maule, Talca, Chile

**Keywords:** educational neuroscience, executive function, measurement, inhibitory control abilities, interventions

The aim of this Research Topic was to promote the continuing growth of educational neuroscience and developmental cognitive science in the field of education. To this end, we present a set of connected publications which examine issues related to measurement and the means by which we might understand individual variation in learners' abilities and behaviors. Through a range of behavioral and neuroscientific methods, these articles provide insights into the cognitive processes and neurocognitive mechanisms important for achieving optimal educational outcomes across different ages, disciplines, and learning settings.

When considering the cognitive processes important for optimal educational outcomes, executive function is often a point of focus due to considerable evidence for the fundamental part it plays in learning processes. Though typically examined in relation to numeracy and literacy, there is now a growing focus on the role this ability, or set of abilities, plays in science learning. For instance, Varma et al. contributed to this Research Topic with an empirical study investigating the role of executive function in scientific reasoning in young adolescents. Using a range of executive function measures, they found that updating (the ability to replace outdated information with new, relevant information) strongly predicted science achievement whilst cognitive flexibility (the ability to adapt thinking based on task demand) held a weaker relationship. Notably, these results extend past work by indicating that executive function plays a role in the acquisition of new concepts as well as in the suppression of erroneous ideas via inhibitory control. Similarly, Park et al. examined executive function in learning of science concepts in the context of interleaved instruction, comparing this to blocked instruction among adolescents. Students who received interleaved instruction on geological concepts performed better on a subsequent test on the subject matter than those who received blocked instruction. Importantly, though, they found that executive abilities were more predictive of learning in the interleaving group compared to the blocked group, suggesting a key interaction between pedagogy and individual ability. Together, these findings contribute to our understanding of the type of instruction and support students might require to aid understanding of science concepts and subsequent reasoning.

Intervention programmes can be developed from such studies to help students who struggle in the classroom. However, the implementation of such programmes in schools can be complex, and Song et al. have identified several challenges and opportunities regarding the translation and implementation of education-based cognitive training programmes. They looked specifically at the effectiveness of working memory training and found that one issue is the disconnect between general theoretical mechanisms and practice. They recommend that training method, setting, and individual differences of both trainees and implementers all be considered as contributing factors to the effectiveness of training programmes. They also encourage acknowledgment of the collaboration between educators and researchers in implementing such interventions. Rogers et al. suggested similar methodological flexibility when studying the role cognitive processes and neurocognitive mechanisms play in learning, using quantitative and qualitative methods to explore the links between executive control and creativity in primary school children. Using quantitative methods, they measured children's executive control and creative thinking capacities, while qualitative findings demonstrated *how* children use executive control in creative tasks. This facilitated an understanding that children can achieve similar results in creative tasks with a great deal of variation in how much executive control is used. The studies by Rogers et al. and Song et al. both contribute to a growing call for increased ecological validity in educational neuroscience research.

In line with this, Schroer et al. assessed executive function in 2-to-3-year-old children using ecologically valid methods. Children were asked to build a tower using Duplo/Lego blocks whilst adhering to two rules (height of tower and alternation of block colors). It was found that performance on a standardized measure of inhibition was linked to adherence to the height rule, whereas working memory was linked to performance on the color rule. This simple and ecologically valid measure could provide an accessible method of measuring executive abilities in very young children. Related to this, Richland and Zhao raised the issue that the refinement of executive function measures for the purpose of understanding complex cognition has resulted in the tasks being removed from everyday activity. Consequently, they argue that the absence of context in executive function measures places a requirement on the participant to use abstract reasoning to complete the task. This, in turn, might align with abilities fostered in Western, Educated, Industrialized, Rich, Democratic (WEIRD) cultures and not across all contexts (as should be their purpose). They also posit that relational attention and reasoning should be part of what we refer to as executive function, as this would be more reflective of what is actually being assessed. Furthermore, the importance of including attention in measurement batteries is supported by Godwin et al. in their study of maths proficiency in adults. They measured relationships between performance on fraction arithmetic, inhibitory control, and attention to relevant components of the fraction. By using heat maps to assess attentional focus, they found that both inhibitory control *and* regulation of attention to strategy-relevant fraction components were linked to the accuracy of the sums.

There are other factors to consider when examining student needs in relation to optimal learning outcomes, and Nieuwenhuis et al. have presented evidence for the potential protective effect of a growth mindset on school burnout symptoms in this population. A growth mindset is the concept that abilities important in academic performance are malleable. The article provides evidence that a growth mindset was linked to reduced signs of burnout in adolescent school children after controlling for academic track record and socioeconomic status. The authors found no evidence for physiological resilience as a mechanism underlying this link, suggesting that cognitive processes should be central in interventions aimed at burnout in adolescents.

Together, these studies demonstrate ways in which a focus on key processes and more refined methodologies might allow educational neuroscience and developmental cognitive science to go further in offering novel insights into the cognitive processes and neurocognitive mechanisms involved in learning from early childhood to adulthood.

## Author contributions

RG: Conceptualization, Writing – original draft. RF: Writing – review & editing. CR: Writing – review & editing. AT: Writing – review & editing.

